# The Inner Shell Spectroscopy beamline at NSLS-II: a facility for in situ and operando X-ray absorption spectroscopy for materials research

**DOI:** 10.1107/S160057752200460X

**Published:** 2022-05-26

**Authors:** Denis Leshchev, Maksim Rakitin, Bruno Luvizotto, Ruslan Kadyrov, Bruce Ravel, Klaus Attenkofer, Eli Stavitski

**Affiliations:** a National Synchrotron Light Source II, Brookhaven National Laboratory, Upton, NY 11973, USA; b Material Measurement Science Division, Material Measurement Laboratory, National Institute of Standards and Technology, Gaithersburg, MD 20899, USA

**Keywords:** beamline, monochromator, X-ray absorption spectroscopy

## Abstract

The Inner Shell Spectroscopy (ISS) beamline on the 8-ID station at the National Synchrotron Light Source II (NSLS-II), Upton, NY, USA, is a high-throughput X-ray absorption spectroscopy beamline designed for in situ, operando, and time-resolved material characterization using high monochromatic flux and scanning speed. This contribution discusses the technical specifications of the beamline in terms of optics, heat load management, monochromator motion control, and data acquisition and processing. Results of the beamline tests demonstrating the quality of the data obtainable on the instrument, possible energy scanning speeds, as well as long-term beamline stability are shown. The ability to directly control the monochromator trajectory to define the acquisition time for each spectral region is highlighted. Examples of studies performed on the beamline are presented. The paper is concluded with a brief outlook for future developments.

## Abstract 2

Technical specifications of the Inner Shell Spectroscopy beamline at the National Synchrotron Light Source II of Brookhaven National Laboratory are described.

## 1. Introduction

X-ray absorption spectroscopy (XAS) is one of the most powerful and widespread synchrotron-based methods with high-profile applications in biology, chemistry, condensed matter physics, and materials science (DeBeer, 2014 ▸; Garino *et al.*, 2014 ▸; Boscherini, 2013 ▸; Frenkel, 2012 ▸; Van Bokhoven & Lamberti, 2015 ▸; Feiters & Meyer-Klaucke, 2020 ▸; Fang *et al.*, 2021 ▸; Timoshenko & Roldan Cuenya, 2021 ▸). X-ray absorption spectra comprise X-ray absorption near-edge structure (XANES) and extended X-ray absorption fine structure (EXAFS) which deliver element-specific information on the local electronic structure and coordination environment, respectively, making it a superior tool for establishing structure–function relationships in various systems regardless of their phase or crystallinity (Rehr & Albers, 2000 ▸; Lee *et al.*, 1981 ▸). The recent advent of third- and fourth-generation X-ray light sources enabled XAS collection times to be shortened to seconds and less thanks to the increased X-ray flux, further enhancing the technique’s temporal resolution (Van Bokhoven & Lamberti, 2015 ▸). Compatibility of XAS with multiple designs of reactors and other complex environmental cells has allowed materials to be characterized during chemical reactions under working conditions, *i.e.* in situ and operando (Singh *et al.*, 2010 ▸; Fabbri *et al.*, 2017 ▸). This advancement has enabled deciphering complex chemical processes by providing element-specific information into the reaction kinetics in multicomponent systems, which has led to new insights in catalysis and battery research (Frenkel *et al.*, 2011 ▸; Croy *et al.*, 2011 ▸; Chen *et al.*, 2019 ▸). Further improvement of time resolution by coupling XAS with pump–probe laser setups has enabled observation of transient species in photo-driven reactions in pico- and femtosecond regimes (Chen *et al.*, 2014 ▸; Shelby *et al.*, 2016 ▸). Additionally, with high flux it became possible to run standard XAS experiments at the large scale allowing for high-throughput screening campaigns in materials search and characterization (Shi *et al.*, 2011 ▸). Finally, high-flux XAS stations are capable of robust characterization of ultra-dilute systems, opening up new avenues for environmental research where species of interest reach mass fractions of below the 1 mg kg^−1^ level (Gräfe *et al.*, 2014 ▸; Proux *et al.*, 2017 ▸; Manceau *et al.*, 2015 ▸) or in single-atom catalysis (Wang *et al.*, 2018 ▸; Li *et al.*, 2019 ▸; Zhang *et al.*, 2020 ▸). Overall, ever-increasing demand for XAS experiments calls for further development of advanced instrumentation capable of rapidly collecting high-quality data.

Traditionally, XAS data collection is executed by step-wise motion of the monochromator through the relevant energy range, using a step size chosen to balance competing factors such as the desired energy resolution, intrinsic instrumental resolution, and the time constants governing the process under study. Regardless of the step size, the scan routine typically allows for settling time after each move, a fraction of a second, increasing the overhead, measurement time, and radiation dose on the sample, potentially resulting in photoinduced sample damage. In the last two decades, two different strategies have been developed in parallel allowing for a substantial reduction of the measurement time down to seconds or faster. The first strategy is based on the energy-dispersive XAS (EDXAS) approach, where the XAS measurement is carried out by recording the transmitted intensity of a spatially dispersed white or pink X-ray beam using an area detector (Baudelet *et al.*, 2011 ▸; Poo-arporn *et al.*, 2012 ▸; Pascarelli *et al.*, 2016 ▸). This allows an entire XAS spectrum to be recorded in a single exposure without moving any mechanical components, which substantially simplifies the data acquisition. Thanks to the high temporal resolution achievable with this method, it has become popular in the fields of dynamic compression and sub-millisecond catalysis (Marini *et al.*, 2014 ▸; Kong *et al.*, 2012 ▸). Although highly effective from the data collection point of view, the EDXAS method imposes relatively strict requirements on the sample under investigation, as it must be collected in transmission mode and its application is limited to only concentrated samples. Furthermore, the morphology of the sample has a strong impact on the results of EDXAS experiments as samples producing strong small-angle scattering patterns may distort the recorded spectra (Pascarelli *et al.*, 1999 ▸). These shortcomings of the EDXAS method can be addressed by adapting the so-called turbo-XAS approach. Here, the data are acquired sequentially by moving a slit across the incident polychromatic beam, monochromatizing it and allowing for fluorescence measurements of dilute samples (Pascarelli *et al.*, 1999 ▸; Nagai *et al.*, 2008 ▸).

The second strategy, the quick-EXAFS (QEXAFS) approach, is based on rapid continuous scans through the relevant energy range by oscillating the monochromator around the selected Bragg angle. QEXAFS motion schemes have been realized with traditional monochromators based on goniometers (Lee *et al.*, 1998 ▸; Oji *et al.*, 2012 ▸; Nikitenko *et al.*, 2008 ▸; Clausen *et al.*, 1993 ▸; Dent *et al.*, 2013 ▸; Martel *et al.*, 2012 ▸; Uruga *et al.*, 2009 ▸; Prestipino *et al.*, 2011 ▸), piezoelectric tilt-tables (Frahm *et al.*, 2009 ▸; Lützenkirchen-Hecht *et al.*, 2001 ▸; Bornebusch *et al.*, 1999 ▸; Richwin *et al.*, 2001 ▸), and cam-shaft driven designs with the latter demonstrating the best performance in terms of stability, motion reproducibility, and scanning speed (Fonda *et al.*, 2012 ▸; Frahm *et al.*, 2004 ▸; Stötzel *et al.*, 2010 ▸; La Fontaine *et al.*, 2013 ▸). While these designs improve the temporal resolution of XAS experiments down to the millisecond regime, they usually allow only sinusoidal motion around the absorption edge of interest. Investigation of several absorption edges requiring fast energy changes on the order of several keVs requires more advanced designs, such as coupling two monochromators within the same cage (Fonda *et al.*, 2012 ▸). Most modern developments in QEXAFS methodology employ servo, or torque, motors that are directly coupled to the monochromator crystal cage and allow for simpler mechanical designs. Further, monochromators are improved in terms of heat management by means of indirect or direct cooling systems making them compatible with insertion device beamlines. The latter achieves monochromatic flux above 10^13^ photons s^−1^ at the sample hence providing best-in-class flux for QEXAFS measurements (Nonaka *et al.*, 2012 ▸; Bornmann *et al.*, 2019 ▸; Caliebe *et al.*, 2019 ▸; Müller *et al.*, 2015 ▸, 2016 ▸).

The Inner Shell Spectroscopy (ISS) beamline described herein belongs to a modern class of QEXAFS beamlines using an insertion device as a photon source and employing a directly liquid nitro­gen (LN_2_) cooled rapid scanning monochromator driven by a servo motor. The beamline X-ray source is a damping wiggler which covers a wide variety of edges in the hard X-ray regime between Ti and I *K*-edges, 4966 eV and 33169 eV, respectively. Unlike traditional QEXAFS monochromators allowing only oscillating trajectories, the ISS monochromator mechanics allow custom trajectories providing better control over the acquisition time at each spectral region. The asynchronous approach to data acquisition implemented here provides particular utility for time-resolved experiments. The beamline is also compatible with a variety of sample environments, making it suitable for experimentation in the fields of materials science, catalysis, and condensed matter physics. In this paper, we describe the technical specifications of the beamline and showcase some of its capabilities.

## 2. Description of the beamline

**Figure 1:**
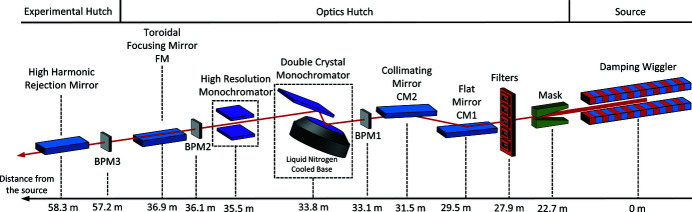
Optical scheme of the Inner Shell Spectroscopy beamline at NSLS-II. BPM denotes beam position monitor.

The ISS beamline is located at Sector 8-ID of the National Synchrotron Light Source II (Upton, NY, USA) – a 3 GeV storage ring with designed operating current of 500 mA. The optical scheme of the beamline is shown in Fig. 1 ▸. The beamline source is a 7 m-long damping wiggler (DW) comprising 75 periods with 90 cm period length. The DW has a critical energy of 11 keV and *K* parameter of 15.8. The DW is a part of the accelerator lattice designed to reduce the beam emittance; DWs are currently integrated in the NSLS-II and PETRA-III accelerators (Tischer *et al.*, 2008 ▸; Tanabe *et al.*, 2014 ▸). The DW produces 60 kW of radiation power of which only 5.7 kW, confined in a 1 mrad [horizontally (h)] × 0.1 mrad [vertically (v)] fan, is accepted by the entrance mask of the beamline (Choi & Willeke, 2012 ▸). Following the mask, the beam enters the first optics enclosure hutch of the beamline. The first component of the beamline is a set of pyrolytic graphite filters used to remove the low-energy radiation from the spectrum to minimize the heat load on the downstream components. The filter assembly is translated vertically to select a filter of thickness between 25 µm and 2 mm appropriate to the energy range required for the experiment.

The following optical component is the dual high-heat-load/collimating mirror system. First, the beam is reflected upwards with a Si flat high-heat-load mirror (CM1) which is directly water-cooled (Tonnessen *et al.*, 1996 ▸). This mirror has three stripes: bare Si, Pt-coated, and Rh-coated. The second Si collimating mirror (CM2) is cooled indirectly with the cooling manifold coupled to silicon by means of Ga–In eutectics. CM2 has Rh-coated and Pt-coated stripes and is bent into a cylindrical shape using a piezo actuator to deliver parallel beam to the monochromator. For both mirrors, the stripes have an optically useful length and width of 1300 mm and 40 mm, respectively, and the metal coatings are deposited on a 50 nm-thick Cr adhesion layer. Both mirrors are placed in a single vacuum tank to facilitate the alignment of radiation protection components. The nominal beam incidence angle on both mirrors is 2.2 mrad. Each mirror is equipped with two horizontal motion stages to switch between stripes and three vertical stages for alignment.

**Figure 2:**
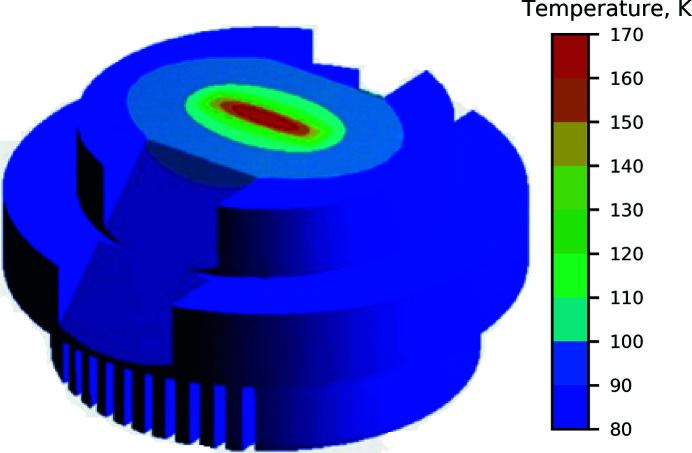
Design of the ISS DCM first crystal along with the finite element analysis of the temperature distribution during normal operations. The top of the crystal is the diffracting surface, while the back of the crystal contains the channels used for the LN_2_ cooling. The temperature map is given for the maximum heat load of 2.2 kW.

Following the mirror system, the ISS Si(111) double-crystal monochromator (DCM) is installed. The first crystal is directly LN_2_-cooled. The cryocooler is equipped with a custom Barber–Nichols pump to provide a peak LN_2_ flow rate of 15 L min^−1^, allowing to dissipate a heat load of up to 2.2 kW with peak power density of 7 W mm^−2^ (Mochizuki *et al.*, 2018 ▸). The first crystal has a cylindrical shape with a 77 mm diameter of its diffracting surface. Cooling channels are cut into the back of the crystal to improve heat exchange with the LN_2_ supply. The maximum temperature achieved at the diffraction surface at the operating heat load is calculated to be 168.4 K which rapidly decays towards the core of the crystal to the value of 83.5 K (Fig. 2 ▸). The associated crystal deformations perpendicular to the crystal surface are estimated to be negligible compared with the Si(111) Darwin width (Mochizuki *et al.*, 2018 ▸). The second crystal is rectangular and is cooled indirectly through clamping to an LN_2_-cooled manifold with a copper foil stack. The second crystal is equipped with a linear translation stage for adjusting the distance between the crystals and therefore the height of the beam at the exit from the DCM. The two-crystal assembly is mounted on a rotary table actuated by an on-axis servo motor to allow for a Bragg angle in the range 3.2–26°. The motor, installed outside of the vacuum chamber, is coupled to the in-vacuum rotary table through a ferrofluidic feedthrough. Energy scans are executed in a pseudo-channel cut geometry. A piezo actuator for the second crystal pitch is used to keep two crystals parallel during the scans with the aid of a feedback loop as described below. A second piezo actuator adjusts the roll angle of the crystal assembly. The specifics of the DCM motion control are described below.

An additional high-resolution monochromator with a channel-cut Si(220) crystal is installed downstream of the DCM and is designed to move in tandem with the DCM to provide improved X-ray energy resolution for XAS measurements. The crystal is mounted on a goniometer and rotated by an on-axis servo motor in the range between 5.3 and 45°. This optical element is currently under commissioning at the beamline and, while the general motion strategy will be reminiscent of that of the DCM, the details of the coupled motion of two monochromators will be published elsewhere.

The focusing mirror system (FM) refocuses the beam to the sample position with two available mirrors, one with a Rh coating and the second with a Pt coating, deposited on a 50 nm-thick Cr adhesion layer. The optically useful length and width of each mirror is 1300 mm and 40 mm, respectively. The sagittal bending radius of each mirror is 62.5 mm. A piezoelectric actuator is used to bend the mirrors into a toroidal shape with a nominal tangential bending radius of 21 km. The motion system of the mirror is also equipped with a set of stages similar to that used with the collimating mirrors for alignment and switching between two stripes. The angle between the toroidal mirror and the beam is set to 2.2 mrad. In addition to beam focusing, the mirror also minimizes the vertical beam movement at the sample position caused by changes in the beam height at the exit from the DCM during energy scans.

The beamline is equipped with optical beam position monitors BPM1-3 placed between major optical components to aid beamline alignment and diagnostics (Fig. 1 ▸). The BPMs use nitro­gen-doped CVD diamond screens [75 mm (h) × 36 mm (v)] oriented at 45° with respect to the beam. The BPM1 diamond has a thickness of 50 µm and is braised to a copper water-cooling block. The BPM2-3 diamonds are 100 µm thick. The BPM assemblies are mounted on pneumatic actuators allowing insertion and retraction on demand. Allied Vision Prosilica cameras are used to visualize the beam.

The final optical component of the beamline is the high-harmonics-rejection mirror system installed in the endstation hutch. The system consists of three mirrors, bare Si, Rh-coated, and Pt-coated, each having optically useful length and width of 600 mm and 80 mm, respectively. The mirror system is equipped with a horizontal and two vertical stages for switching between mirrors and adjusting the pitch angle. An additional in-vacuum actuator is available for fine pitch adjustment. The beam incidence angle on the mirror is set to 2.5 mrad. The mirror is selected based on the experimental needs: the Si mirror is used for measurements at energies below 12 keV; the Rh mirror is used in the range between 12 and 23 keV; the Pt mirror is used beyond 23 keV. The beam size at the sample position is 1 mm × 1 mm (FWHM) and the flux is 5 × 10^13^ photons s^−1^ at 12 keV as estimated from the ion chamber current.

### 2.1. The ISS monochromator motion

The ISS DCM motion system is designed to perform scans at a state-of-the-art speed of more than 1000 eV s^−1^, while moving the crystals smoothly and reproducibly along with the ∼50 kg mechanical load of the goniometer table and the LN_2_ cooling system. The motion control system for the DCM is based on a real-time, embedded, Linux-based Power PMAC controller (Delta Tau systems). The choice of this motion controller was dictated by the need to generate motion trajectories with high sampling rate (16 kHz), and the network capability to upload a user-calculated trajectory for the desired experimental energy profile. The ISS DCM motion system uses a 2.5 kW rotary direct drive AC brushless servomotor (Nikki Denso NMR-BFFCA2A-252A) driven by a Nikki Denso NCR servo driver. The Bragg axis position is registered using dual Renishaw RESM rotary encoders of diameter 413 mm and the TONiC readhead with 2000× interpolator, with a resulting encoder resolution of 50 nrad.

**Figure 3:**
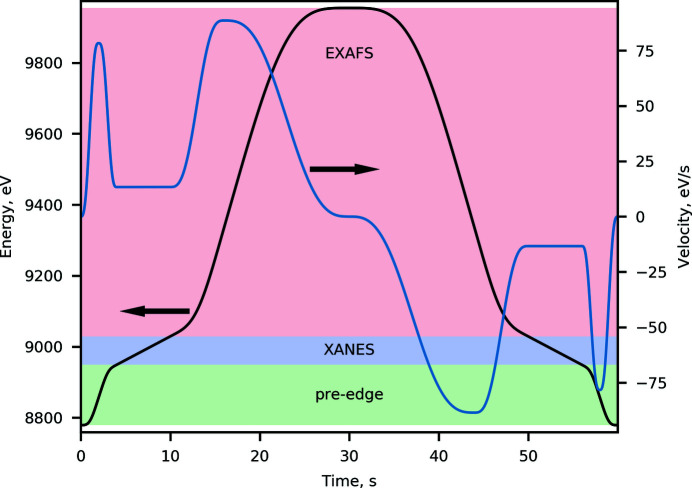
Example of a motion trajectory designed at ISS for the Cu *K*-edge at 8979 eV. Energy and velocity profiles are shown in black and blue, respectively.

As mentioned before, traditional QEXAFS monochromators exhibit oscillatory motion over a desired energy range. As different regions of the XAS scan require different signal-to-noise ratios and sampling densities to yield meaningful results, the accelerations and speeds in a sinusoidal trajectory do not match the required acquisition times for each region. At ISS, the Power PMAC controller enables custom motion profiles that closely match the spirit of a traditional XAS scan. As an example, the motion profile along with the corresponding velocity profile around the Cu *K*-edge is shown in Fig. 3 ▸. Here, we have implemented a trajectory where the monochromator quickly scans through the pre-edge region using a quarter-sine profile, slows down and moves linearly in energy in the XANES region, and then follows a quarter-period sine profile throughout the EXAFS region. The motion is then reversed to return to the starting point and to be repeated as needed. This type of trajectory was designed to eliminate the non-continuities in the speed and acceleration making it compatible with the heavy mechanical load of DCM internal components. The example shows the trajectory executed on the time scale of 1 min; however, faster scanning is possible as shown below. The scanning speeds and inflection point positions can be further adjusted to fine-tune the trajectory to optimize the time spent at specific spectroscopic regions.

To execute a trajectory, a list of desired encoder positions at the servo rate (16 kHz) is loaded into the controller as an ASCII file through FTP. Up to ten trajectories can be stored in the Power PMAC flash memory which allows quick switching between different absorption edges. At execution, the trajectory values are copied into the controller random access memory (RAM) and at each servo interrupt the motion controller utilizes the next position value from the trajectory as the commanded position for the PID (proportional-integral-derivative) controller to act upon.

Maintaining the parallel orientation of DCM crystals during Bragg axis rotation is necessary for successful execution of energy scans. However, due to the thermal and mechanical distortions of the second crystal caused by clamping to the cooling system, the parallel alignment can be lost during a scan. To address this issue, a feedback loop was implemented at ISS and is based on the following approach. Deviations from the crystal parallelism cause variation in the beam angle at the DCM exit which translates into variation of the vertical beam position on the sample. Therefore, DCM crystal parallelism can be maintained by correcting the second crystal pitch such that the vertical position of the beam remains constant in the vicinity of the sample during an energy scan. The implemented feedback mechanism tracks the beam height using BPM3, located upstream of the sample, and corrects the second crystal pitch during a scan using a PID control loop.

### 2.2. The endstation

The ISS endstation is designed to support experiments in catalysis, battery cell, and materials research. The sample positioning is executed with heavy-duty translation stages capable of carrying large sample cells in the beam while providing an ease-of-access to the setup for users (Qu *et al.*, 2021 ▸). The beamline supports a sophisticated gas delivery system that can supply various gases, such as H_2_, O_2_, CO, CO_2_, He, and Ar, and flow rates up to 100 cm^3^ min^−1^. The gas handling systems can be further combined with the heating cells that can achieve temperatures up to 1100–1200°C.

To accommodate a wide variety of spectroscopic experiments, ISS is equipped with several detectors. The detection system of the beamline includes the standard set of ionization chambers (ICs) and a silicon detector, which are used to measure sample and reference transmission signals, as well as sample fluorescence. The ICs are manufactured in-house and have lengths of 17 cm, 30 cm, and 17 cm for incident, transmitted, and reference channels, respectively. The ICs are operated at atmospheric pressure and are filled with varying ratios of He and N_2_ gases ranging from 5:1 at 5000 eV to 2:5 above 20000 eV. The total gas mixture flow is ∼130 cm^3^ min^−1^. A high-voltage module EHC 8240 (Wiener Power Electronics GmbH) is used to bias the ICs in the 1000–1700 V range, depending on the energy range. The gas composition and voltages can be controlled remotely for ease-of-use at different energies. Total fluorescence yield signal is measured with a passivated implanted planar silicon detector (PIPS, Mirion Technologies) with a 300 µm-thick Si chip and an active area of 30 cm^2^. A set of filters and Soller slits are available to be used with the PIPS detector to suppress elastic and Compton scattering. Each IC and PIPS is paired with a remotely controlled DHPCA-100 transimpedance amplifier (FEMTO Messtechnik GmbH) with its output voltage read by the data acquisition system as described below. To aid fluorescence measurements from dilute systems, ISS is equipped with a silicon drift diode (SDD) detector comprising four VITUS CUBE H50 elements each paired with VIAMP preamplifiers (Ketek GmbH) and coupled with an Xspress 3X readout electronics module (Quantum Detectors Ltd). A Pilatus 100k is also available for experiments requiring position-sensitive detection, such as diffraction. In addition, both the SDD and Pilatus 100k detectors can be paired with a crystal analyzer to perform high-energy-resolution X-ray emission experiments. The arrangement of the detectors is highly flexible and can accommodate a broad spectrum of sample environments.

### 2.3. Microfocusing capabilities

**Figure 4:**
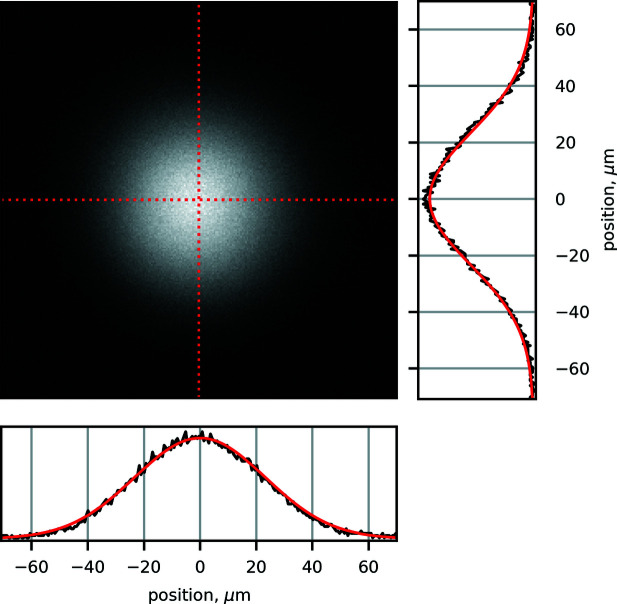
Beam profile produced by a polycapillary lens optic installed at the ISS beamline at 12 keV. The cuts through the profile image are shown in red while the right and bottom panels show the corresponding intensity distributions along with Gaussian fits in black and red, respectively. The FWHM of the beam is 55 µm vertically and horizontally.

The beamline offers an option to deliver a focused beam on the sample by means of a polycapillary lens (PCL). The beamline is equipped with several PCLs (XOS Instruments and Institute for Scientific Instruments GmbH), with a working distance of 10 mm to 30 mm. A six-axis manipulator is used to align the PCL with the beam to maximize the efficiency and focal size. The Gaussian beam profile obtained using the PCL has a FWHM of 55 µm vertically and horizontally at 12 keV as shown in Fig. 4 ▸. Other optics are also available to focus beams down to 100 µm. The transmission of the optic at 12 keV is approximately 25% and as low as 10% at energies above 25 keV. The PCL can be used in experiments on samples with large grain size to improve the quality of the data, as well as high-energy-resolution spectroscopy applications currently under development on the beamline. We note that during early stages of ISS development an internal ray-tracing study demonstrated that ∼100 µm focus, required for high-energy-resolution spectroscopy applications, cannot be achieved with more standard approaches, such as Kirkpatrick–Baez mirrors, due to the high divergence of the beam produced by the 7 m-long DW source.

### 2.4. Beamline controls

**Figure 5:**
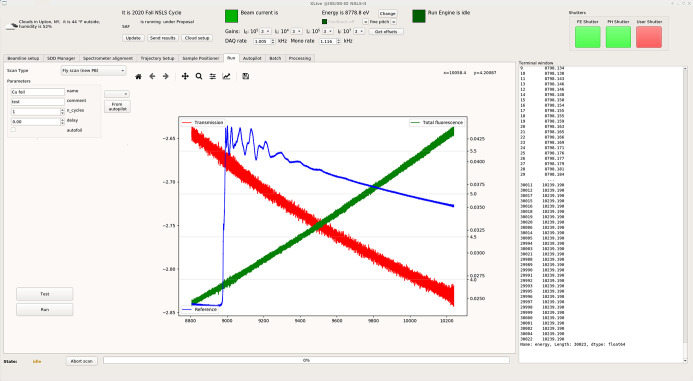
Screenshot of *XLive* software showing interpolated data from a Cu foil reference in the main plot. The central plot shows the raw transmission, fluorescence, and reference absorption signals that are obtained before binning, which is explained in the main text. Since only reference Cu foil was put into the beam, the reference channel is the only one showing a signal.

The beamline controls are implemented using several software packages. Basic communication between hardware devices is controlled via an EPICS interface, whereas experimental control and data collection are performed using the *Ophyd* (https://blueskyproject.io/ophyd) and *Bluesky* (https://blueskyproject.io/bluesky) libraries (Allan *et al.*, 2019 ▸; Arkilic *et al.*, 2017 ▸). This controls implementation allows scripts to execute scientific experiments in the Python language, facilitating complex measurement routines. The high-level beamline controls are carried out using *Controls System Studio* (*CSS*, https://controlsystemstudio.org/) and Python/Qt-based graphical user interfaces (GUIs). These controls allow users to access all the relevant beamline components to perform the experiments. The package available on the beamline, *XLive* (Luvizotto *et al.*, 2017 ▸), is a tool actively developed by the beamline staff and is available publicly through GitHub (https://github.com/NSLS-II-ISS). *XLive* provides users with straightforward access to the beamline setup and data acquisition configuration, DCM trajectory settings, sample positioning, and experimental setup parameters (Fig. 5 ▸). One of the *XLive* features is the ability to set up highly automatic scanning routines that can acquire data on large batches of samples and perform sets of different scans on multiple absorption edges. Overall, *XLive* is developed in order to be an easy-to-use tool for users with all levels of XAS expertise, as well as allowing users setting up highly customized and complex experimental strategies.

## 3. Data acquisition system

**Figure 6:**
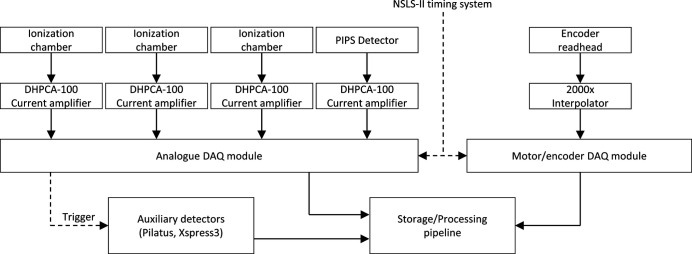
Data acquisition scheme implemented at the ISS beamline. The dashed lines represent the flow of timing signals and solid lines represent the flow of experimental signal/data streams.

A novel data acquisition (DAQ) architecture was developed at ISS which relies on asynchronous continuous collection of relevant data streams. Along with the data, each device in the distributed DAQ system has an event receiver which uses NSLS-II timing information to independently timestamp each measured data point with 8 ns resolution (Fig. 6 ▸). The data streams are aligned in the post-processing to produce conventional scans (*e.g.* detector versus monochromator or other motor position). This approach has the unique advantage that the DAQ modules can operate independently and permits the system to scale indefinitely allowing the execution of highly complex experiments as the timestamping is used to correlate events from the multiple sources.

Analog detector signals (ion chambers, diodes) are captured using an FPGA-based module which was developed at NSLS-II based on the electron beam position monitor cell controller and is internally referred to as Analog Pizza Box or APB. It employs a Xilinx Zynq processor with an ARM A9 dual core processor, Gigabit Ethernet, 64 Gbytes of FLASH memory via an SD card as well as six SFP slots for time receiver input and future high-speed interconnect. The module runs Debian Linux and the EPICS IOC constituting a complete standalone system. It has an 8-channel/18-bit analogue-to-digital converter (ADC) capable of sampling at rates up to 1 Mbps, 8-channel/18-bit digital-to-analogue converter (DAC), as well as eight general purpose TTL inputs/outputs. The ADC sample rate can be locked to the NSLS-II revolution frequency if needed so that samples across modules are completely synchronized. The FPGA provides additional averaging and filtering capabilities, for improving the signal-to-noise ratio of the measured signals.

Motor positions are captured by recording the real-time quadrature incremental encoder readout on the relevant motion axes. The encoder module leverages an NSLS-II BPM Digital Front End (DFE) board with Virtex-6 FPGA, Gigabit Ethernet and SFP transceivers. A MicroBlaze soft processor is implemented in the FPGA for data transportation using Xilinx lwIP library. The module supports four identical, individually isolated channels with encoder input capturing (RS-422A, up to 10 MHz). The encoder signal is re-broadcast to the motion controller if necessary. Position filtering is optional for input encoder signals to avoid data overflow due to the limitation of the TCP/IP stack bandwidth. Auxiliary digital TTL I/O is implemented for external triggering by recording input transition and toggling the output as commanded by software.

Other detectors (Xspress 3X readout system, Pilatus 100k area detector, and others) are integrated into the ISS DAQ systems by means of a series of TTL triggers produced by the APB device (Fig. 6 ▸). Each pulse in the pulse train is captured together with the timestamp. Based on the time information associated with every frame captured by the detector, the data are correlated with other data streams.

With the open architecture of the asynchronous DAQ system, data from the *in situ* reaction environment can be also captured and correlated with the XAS measurements parameters, allowing for complex experimental schemes (Singh *et al.*, 2018 ▸; Qu *et al.*, 2021 ▸).

### 3.1. Data processing

During the energy scan, the rotary incremental encoder position of the monochromator Bragg angle axis and the detectors signals are being captured by different devices as individual data streams. Thus, the first step in the data processing is to align the data streams onto the uniform time grid, using interpolation. With this step, the energy position and the detector readout are correlated in time. In the next step the data are re-binned onto a conventional XAS grid, *i.e.* large (∼10 eV) energy step in the pre-edge, fine (sub-eV) steps in the XANES region, and *k*-space steps in the EXAFS with *k* = 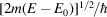 , where *E* is the measured energy, *E*_0_ is the absorption edge position, *m* is the mass of the electron, and 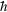 is Planck’s constant. The routine applies a Gaussian filter to the interpolated data. Here, the raw data are represented as [*x*_*i*_; *d*_*i*_], 1 ≤ *i* ≤ *N*, where *x*_*i*_ is the energy position, *d*_*i*_ the detector readout value and the *N* the number of points in a spectrum. The binned data will be [*X*_*j*_; *D*_*j*_] where *X*_*j*_ is a new energy grid. For each *X*_*j*_ point a Gaussian function can be created,

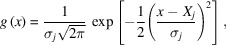

where σ_*j*_ = FWHM_*j*_/2(2ln2)^1/2^ and FWHM_*j*_ = (*X*_*j*_ − *X*_*j*−1_)/2 + (*X*_*j*+1_ − *X*_*j*_)/2.

The binned data elements are then given by the element-wise multiplications

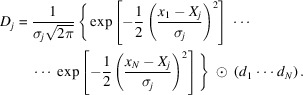

**Figure 7:**
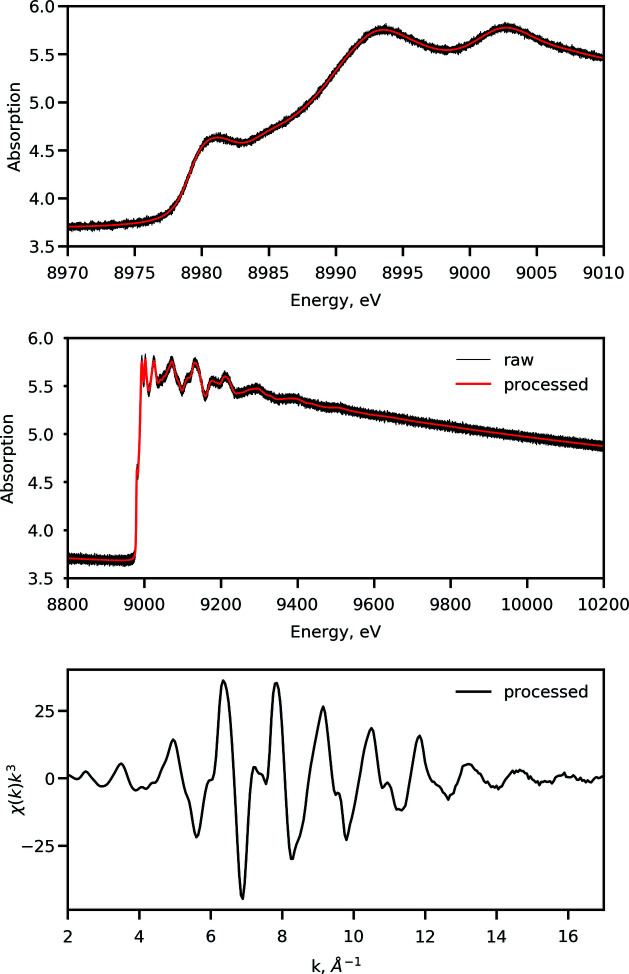
Example of a spectrum measured at ISS on a copper foil at the Cu *K*-edge at 8979 eV using the energy-down portion of the trajectory shown in Fig. 3 ▸. (Top) Zoom on the XANES region of the spectrum. The raw spectrum is overlaid with the processed spectrum obtained using the Gaussian filtering as explained in the text. (Middle) Overview of the entire spectrum recorded using the trajectory. The legend and the ordinate scale are shared between top-left and top-right panels. (Bottom) The *k* ^3^-weighted χ(*k*) function obtained after normalization and background removal of the processed absorption spectrum using *LARCH* (Newville, 2013 ▸).

While the resulting Gaussian filtering may introduce some broadening of the spectral features of interest in the XANES region, its magnitude is on the sub-eV scale, which is smaller than the Si(111) monochromator resolution at a given energy. An example of raw and binned spectra along with the obtained χ(*k*) function is shown in Fig. 7 ▸. This procedure is the default at the beamline; however, the width of the Gaussian filter and the step size in each region can be tweaked to suit specific user data analysis strategies. We note that more advanced filtering options for XAS applications are known from the literature, *e.g.* Butterworth filter (Clark *et al.*, 2020 ▸), and their integration are currently underway.

## 4. Beamline performance

The beamline performance was tested in terms of data quality obtainable using the advanced DCM trajectory control, fast scanning capabilities, and long-term energy calibration stability.

**Figure 8:**
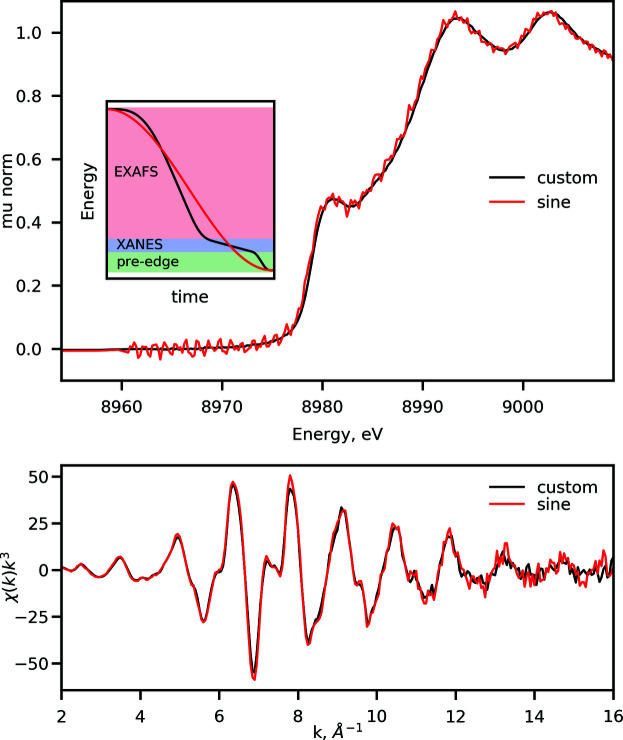
Comparison of the data recorded using the trajectory designed at ISS and sinusoidal motion common at fast scanning XAS beamlines. The data are measured during energy scan down with duration 1 s on copper foil at the Cu *K*-edge at 8979 eV. (Top) Comparison of the processed data in the XANES region. The inset shows a qualitative comparison between the custom trajectory and the sine trajectory with the depiction of the energy regions covered as a function of time. (Bottom) The *k* ^3^-weighted χ(*k*) function obtained after normalization and background removal of the processed absorption spectra measured using the two trajectories.

Fig. 8 ▸ shows a comparison of the data measured using the customized trajectory, shown in Fig. 3 ▸, with the data measured using traditional sinusoidal motion trajectory, typically implemented at quick-scanning XAS beamlines. The comparison was performed using a measurement of the Cu *K*-edge at 8979 eV on a copper foil using second-long trajectories. For comparison purposes we only used the data portion recorded during the monochromator scan ‘down’ in energy, *i.e.* when the energy is decreasing from the highest (∼9955 eV) to lowest (∼8780 eV) energy, covered by the monochromator trajectory. The difference in the quality of the spectra is evident, in particular in the XANES region: since the ISS trajectory is designed to spend ∼3.5 times longer at the XANES region, the signal-to-noise of the data recorded there is higher. On the other hand, the quality of the EXAFS region is similar to that obtained using the sinusoidal trajectory since the time spent in the region is comparable in both trajectories. Overall, compared with the sine motion, the precise trajectory control of the DCM allows more efficient distribution of the experimental time on collection of spectral regions of interest allowing desirable signal-to-noise levels to be obtained faster. This can be further tailored to the specific needs of the experiment at hand and is particularly important in the context of measuring dilute and/or radiation-sensitive samples.

**Figure 9:**
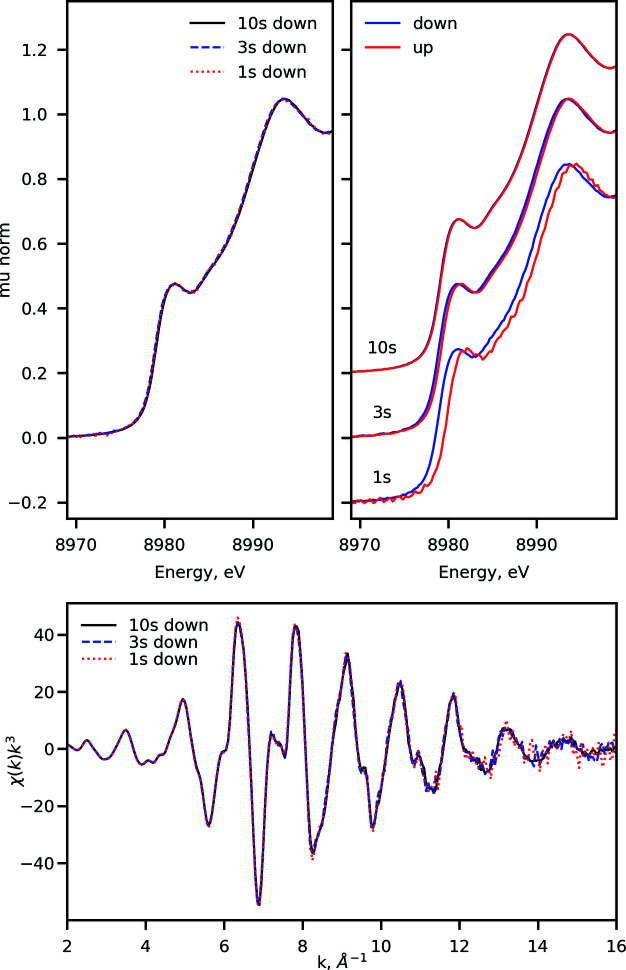
Testing the fast scanning capabilities of the ISS DCM. (Top-left) Comparison of the ‘down’ spectra. The spectra were collected using trajectories with shape shown in Fig. 3 ▸, but with varying durations of 1, 3, and 10 s. (Top-right) Comparison of ‘up’ and ‘down’ spectra recorded with different scanning speeds. (Bottom) The *k* ^3^-weighted χ(*k*) functions measured using different scanning speeds..

Next, we investigate the stability of the repetitive motion of the monochromator as a function of scanning speed. Fig. 9 ▸ shows the results of the fast scanning tests using copper foil at the Cu *K*-edge performed using trajectories similar to the one shown in Fig. 3 ▸ in the 8779–10213 eV range (corresponding to *k* = 16 Å^−1^), but with varying total durations. We find that the spectra recorded during the monochromator ‘energy down’ match well regardless of the scanning speed down to 1 second-long scans. The ‘energy up’ spectra, however, appear to be systematically shifted towards higher energies, possibly due to mechanical wind-up of the Bragg axis. While for relatively slow scans with 10 s duration the energy offset is negligible (<50 meV), faster scans, with (3 and 1) second durations, exhibit shifts on the order of 0.2 and 0.8 eV, respectively. These shifts, however, are found to be highly reproducible and can be therefore corrected for during post-processing; in the future, the DCM crystal cage will undergo redesign to reduce the wind-up effect. We further find that the fastest scanning speed of the monochromator is limited to 3500 eV s^−1^ at the Cu *K*-edge with higher speeds resulting in the loss of the monochromator calibration. With that, the trajectory developed for the ISS DCM scanning EXAFS region up to *k* of 16 Å^−1^ can be executed in as fast as 1 second; shortening of the scanning time will require adjustments to the trajectory to decrease the peak speed and/or shortening of the scanned energy range.

**Figure 10:**
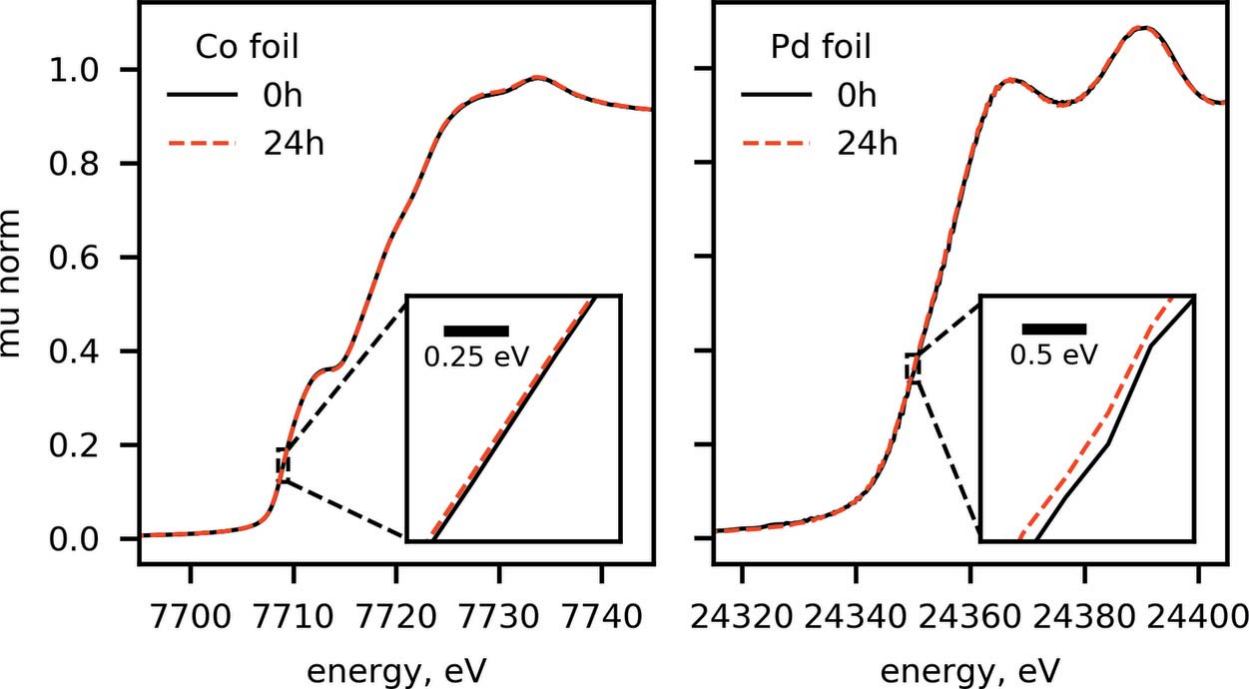
Demonstration of the long-term stability of the energy calibration at ISS at low and high energies. (Left) Comparison of the spectra recorded on Co foil over the span of 24 h. The inset zooms on the data around the edge region where the derivative of the absorption coefficient is at its maximum. The horizontal bar shows the energy scale of the inset. (Right) Comparison of the spectra recorded on Pd foil with a time difference of 24 h. The inset focuses on the edge region of Pd as in the left panel.

The long-term stability of the beamline energy calibration was characterized based on the reproducibility of metal foil spectra obtained during standard user operations involving continuous data recording over a period of 24 h. Fig. 10 ▸ shows the data recorded at lower energies, on cobalt foil at the Co *K*-edge at 7709 eV, as well as at high energies, on palladium foil at the Pd *K*-edge at 24350 eV, recorded during user experiments described in the literature (Liu *et al.*, 2021 ▸; Luneau *et al.*, 2020 ▸; Guan *et al.*, 2020 ▸). We observe that the spectra are reproducible throughout the time frame. For Co, we find that the stability of the edge position is within 0.05 eV, whereas for Pd the edge position remains within 0.2 eV. This indicates the suitability of the beamline for continuous measurements during longer periods of time.

In terms of scientific experiments on real samples, ISS has already contributed to over 100 publications since the beginning of general user operations in 2017. The scope of the studies spans a plethora of subjects, with examples including battery cell research (Lin *et al.*, 2019 ▸, 2020 ▸; Deng *et al.*, 2019 ▸), catalysis (Li *et al.*, 2017 ▸; Xie *et al.*, 2018 ▸; Kauffman *et al.*, 2019 ▸), materials science (Zhang *et al.*, 2019 ▸; Cai *et al.*, 2019 ▸; Juneau *et al.*, 2020 ▸; Liu *et al.*, 2021 ▸), and solid state physics (Liu *et al.*, 2019 ▸, 2020 ▸; Feng *et al.*, 2019 ▸). While the references provided here are not exhaustive, an up-to-date list of publications can be obtained online (https://www.bnl.gov/nsls2/beamlines/publications.php?q=8-ID). Based on the available literature, the beamline has demonstrated the ability to routinely deliver high-quality spectroscopy data on systems with 0.5–5% weight loadings. Challenging low-concentration samples are also amenable to characterization. For example, a recent *in situ* study of TiO_2_ film growth over ZnO nanowires successfully extracted an XANES signal during formation of the first few monolayers of the film (Qu *et al.*, 2021 ▸). The recently commissioned SDD detector has enabled experiments on samples with even lower concentrations, which will be published elsewhere.

## 5. Future outlook

The high flux and the advanced energy scanning capabilities of the beamline open the door to future developments of more advanced spectroscopic photon-in/photon-out techniques. The beamline is currently developing instrumentation for performing high-energy-resolution spectroscopic experiments, such as resonant and non-resonant X-ray emission spectroscopy (RXES, XES) and high-energy-resolution fluorescence detected (HERFD) XAS. These spectroscopic probes provide complementary insights into the electronic structure of materials. For example, HERFD XAS allows to measure absorption spectra with resolution better than core-hole broadening (Glatzel *et al.*, 2013 ▸; Lafuerza *et al.*, 2020*a* ▸; Asakura & Tanaka, 2021 ▸); *K*β XES of 3*d* metals probe their spin state (Vankó *et al.*, 2006 ▸, 2013 ▸; Lafuerza *et al.*, 2020*b* ▸); valence-to-core XES is highly sensitive to local ligand environment of the absorbing atoms (Gallo & Glatzel, 2014 ▸; Pollock & Debeer, 2011 ▸; Pollock & DeBeer, 2015 ▸); and RXES provides detailed information on valence orbitals of the absorbing atom offering insights into chemical bonding and oxidation states in highly covalent systems (Baker *et al.*, 2017 ▸; Castillo *et al.*, 2021 ▸). The beamline aims to provide these capabilities by developing two spectrometers, based on Johann and von Hamos geometries, for non-dispersive and dispersive measurements, respectively, tailored to a variety of experimental needs. First tests using single-element spectrometers have shown promising results in terms of resolution and count rates. Some of the early results on reference compounds obtained using single analyzer spectrometers are shown in the supporting information. In the future, multi-element analyzer arrangements will be installed at the beamline, further enhancing the sensitivity of the instrument. Further developments in controls and sample handling infrastructure will be performed to accommodate and streamline the experiments in a wide variety of disciplines.

## 6. Conclusions

To summarize, we described the ISS beamline design, data acquisition and processing, and overall performance. The focus has been given to the DCM specifications as it is one of the primary elements of the beamline. Specifically, we highlighted the advantages of using the customized trajectories to control the amount of acquisition time for each spectral region, which represents a promising approach to measuring dilute samples. The beamline is capable of producing high-quality spectra containing both XANES and EXAFS in as short as 1 s while maintaining long-term stability over days. The beamline has been already successful in producing scientific output with the existing capabilities, which lays the foundation for future developments. Currently, the beamline is developing capabilities for photon-in/photon-out spectroscopy for XES and RXES measurements, as well as HERFD XAS spectroscopy. These instruments will complement the existing infrastructure and extend the capabilities of the beamline towards more challenging experiments in catalysis, materials science, and beyond.

## Supplementary Material

Figure S1. DOI: 10.1107/S160057752200460X/ye5015sup1.pdf
