# Supplementary material for: The Inner Shell Spectroscopy beamline at NSLS-II: a facility for in situ and operando X-ray absorption spectroscopy for materials research
Source: J Synchrotron Radiat. 2022 May 26;29(Pt 4):1095–106. doi: 10.1107/S160057752200460X (PMC9255565; doi:10.1107/S160057752200460X)
Supplement: Supplementary file 1 [file s-29-01095-sup1.pdf]

JOURNAL OF SYNCHROTRON RADIATION

Volume 29 (2022)

Supporting information for article:

The Inner Shell Spectroscopy Beamline at NSLS-II: a facility for *in situ* and operando X-ray absorption spectroscopy for materials research

Denis Leshchev, Maksim Rakitin, Bruno Luvizotto, Ruslan Kadyrov, Bruce Ravel, Klaus Attenkofer and Eli Stavitski

S1. Initial high energy-resolution spectroscopy tests at ISS

Here we present some of the first results in high energy-resolution spectroscopy obtained at ISS. Figure S1 shows a high energy-resolution fluorescence detected (HERFD) X-ray absorption spectrum (XAS) obtained on potassium hexachloroplatinate, Kβ and valence-to-core X-ray emission spectra (XES) of several Ti containing compounds, and Kβ (1s3p) resonant XES plane obtained from Co_3O_4. The experimental details are summarized in the Figure caption. Typical spectrometer resolutions obtained in these tests were in the 0.5-1 eV range, as estimated from the full width at half maximum values of the elastic scattering peak.

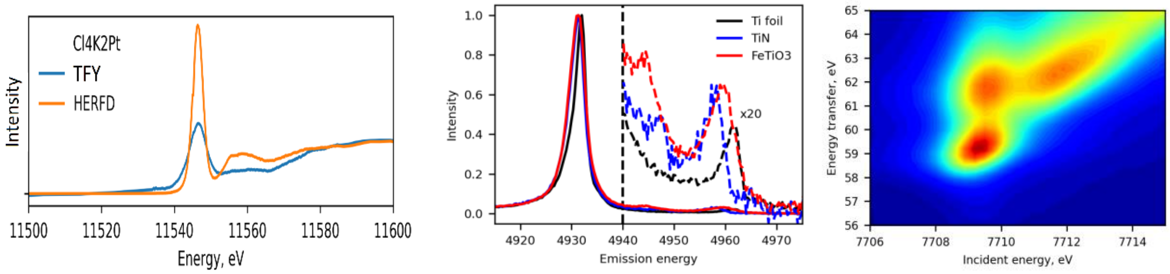

Figure S1 **Figure S1.** Results of initial high-resolution spectroscopy tests performed at ISS. (left) HERFD XAS data on potassium hexachloroplatinate (PtK_2Cl_6) measured in Johann geometry using a spherically curved Si(733) crystal with curvature radius of 1 m. The sample powder was deposited on Kapton tape. The spectrometer was set to the maximum of Pt Lα emission. The energy scans were performed in step mode with 1 s exposure time for each point. (middle) Kβ and valence-to-core XES data collected on neat Ti foil, Ti nitride TiN, and ilmenite $FeTiO_3$ in Von Hamos geometry using cylindrically curved Ge(100) crystal with 0.5 m curvature radius. TiN and ilmenite powders were deposited on Kapton tape. Spectra were collected with incident X-ray energy set to 5200 eV. Total exposure time for each spectrum was within 10-20 min range. (right) Kβ (1s3p) resonant XES data collected on Co_3O_4. The sample was prepared as a mixture of 5 mg of Co_3O_4 mixed with 95 mg of polyethylene glycol pressed into a pellet with 13 mm radius. The data was recorded in Johann geometry using a spherically curved Ge(111) crystal with 1 m curvature radius. The energy scans were performed in step mode with 1 s exposure time per point. In all measurements, Pilatus 100k was used as a detector.
